# Structural variation discovery in the cancer genome using next generation sequencing: Computational solutions and perspectives

**DOI:** 10.18632/oncotarget.3491

**Published:** 2015-03-08

**Authors:** Biao Liu, Jeffrey M. Conroy, Carl D. Morrison, Adekunle O. Odunsi, Maochun Qin, Lei Wei, Donald L. Trump, Candace S. Johnson, Song Liu, Jianmin Wang

**Affiliations:** ^1^ Center for Personalized Medicine, Roswell Park Cancer Institute, Buffalo, NY, USA; ^2^ Department of Gynecologic Oncology, Roswell Park Cancer Institute, Buffalo, NY, USA; ^3^ Department of Biostatistics and Bioinformatics, Roswell Park Cancer Institute, Buffalo, USA; ^4^ Department of Medicine, Roswell Park Cancer Institute, Buffalo, NY, USA; ^5^ Department of Pharmacology and Therapeutics, Roswell Park Cancer Institute, Buffalo, NY, USA

**Keywords:** structural variation, next generation sequencing, cancer genome analysis, somatic mutation

## Abstract

Somatic Structural Variations (SVs) are a complex collection of chromosomal mutations that could directly contribute to carcinogenesis. Next Generation Sequencing (NGS) technology has emerged as the primary means of interrogating the SVs of the cancer genome in recent investigations. Sophisticated computational methods are required to accurately identify the SV events and delineate their breakpoints from the massive amounts of reads generated by a NGS experiment. In this review, we provide an overview of current analytic tools used for SV detection in NGS-based cancer studies. We summarize the features of common SV groups and the primary types of NGS signatures that can be used in SV detection methods. We discuss the principles and key similarities and differences of existing computational programs and comment on unresolved issues related to this research field. The aim of this article is to provide a practical guide of relevant concepts, computational methods, software tools and important factors for analyzing and interpreting NGS data for the detection of SVs in the cancer genome.

## INTRODUCTION

Tumors usually emerge from normal cells by accumulating tissue specific acquired mutations in their genome [1-3]. These somatic mutations are broadly divided into two major categories, Single Nucleotide Variations (SNVs) and Structural Variations (SVs) [4, 5]. SVs were initially defined as genomic alterations that involve DNA segments larger than 1kb [6], then were widened to include any DNA sequence alteration other than SNVs [4, 7]. If somatic acquired SVs alter the expressions of oncogenes or tumor suppressor genes, they could directly contribute to carcinogenesis [8]. For examples, a somatic chromosomal rearrangement fusing two separate genes into a new one such as *BCR-ABL*, *PML-RARα*, *EML4-ALK*, *TMPRSS2-ERG*, or recurrent translocation of genes such as *BRAF* and *CRAF*, is known to be carcinogenic [9]. Therefore, detecting somatic SVs is an essential component in a comprehensive cancer genome analysis.

Traditionally, SVs in the cancer genome can be identified by cytogenetic approaches including fluorescence *in situ* hybridization (FISH) [6]. However, the relatively low resolution and throughput has limited its detection power in complex genomes of epithelial cancers. Microarray-based approaches, including array comparative genomic hybridization (array CGH) and single-nucleotide polymorphism (SNP) arrays, have been widely used in detecting dosage-variant DNA Copy Number Variations (CNVs), a subtype of SVs [10-12]. However, they are not capable of detecting other types of SVs, especially balanced or dosage-invariant DNA sequence rearrangements. Furthermore, they have limited resolution to determine the breakpoint locations. While Sanger sequencing is capable of detecting various types of SVs at the nucleotide resolution, the low throughput and high reagent cost has prevented its adoption in large-scale applications.

The emerging Next Generation Sequencing (NGS) technology provides unprecedented opportunities to systematically screen SVs in the cancer genomes [13]. NGS is a technology that sequences massive amounts of short DNA strands in parallel from randomly fragmented copies of a genome [14, 15]. Comparing to the Sanger-style sequencing, NGS is more financially affordable, less time consuming, and less labor-intensive. When NGS is applied to the whole human genome, it is called Whole Genome Sequencing (WGS). Since WGS can generate multidimensional information for SV discovery in a genome-wide scale, it has become the primary means of interrogating the SVs in recent investigations.

The billions of short reads generated by a WGS run poses unique challenges for SVs detection, and sophisticated computational methods are needed in order to accurately identify the SV events and delineate their breakpoints. Although the NGS technology was only emerging during the past several years, a number of SV detection programs for NGS data have been developed [4, 16-46], with several capable of detecting somatic SVs in cancer genome studies. These programs focus on different subsets of SV types, and use various strategies to detect sequencing signatures or diagnostic patterns indicative of different SV types. As would be expected, each SV caller has its own strength and weakness. In this review, we begin by briefly reviewing the major types of SVs and describing their breakpoint features. We then describe the primary types of NGS signatures that can be used in SV detections, followed by categorizing the existing computational programs into different groups based on the NGS signatures they require. For each group, we first summarize the principles underlying the SV detection, and then comment on the key similarities and differences between each computational program. We continue by providing discussion about the various challenges in somatic SV detection, and conclude with an outlook on the near future of this fast evolving field. The aims of this article are to serve as a timely and practical guide to NGS-based somatic SV studies and to discuss the important factors that researchers need to consider when analyzing NGS data for somatic SV detection.

### SV Types and their breakpoint features

#### SV types

**Figure 1:**
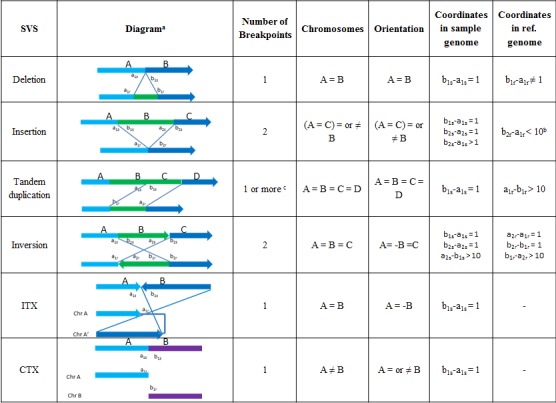
Breakpoint signatures of SVs (a) In each diagram, the up strands are from sample genome, and the lower strand are from reference genome. (b) Depending on the mapping of the inserted strand B, other relationships of coordinates in reference genome can be determined (details not shown). (c) Tandem duplication creates one or multiple breakpoints. NGS is able to detect either 1 (novel tandem duplication) or 0 (non-novel tandem duplication) breakpoint.

**Figure 2:**
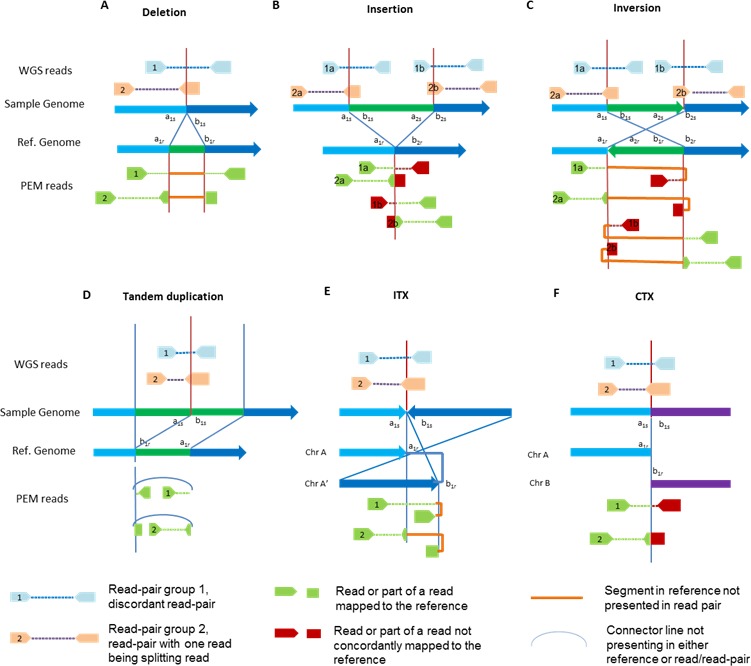
Diagram of SV types and NGS signatures, before and after mapping A) Deletion; B) Insertion; C) Inversion; D) Tandem duplication; E Intra-chromosomal translocation (ITX); F) Inter-chromosomal translocation (CTX).

There are multiple types of SVs [47], but in this review we focus on the six most basic and common ones detected: deletion, insertion, tandem duplication, inversion, intra-chromosomal translocation, and inter-chromosomal translocation (Figures 1 and 2).

*Deletion.* A deletion is an event that occurs when a DNA segment (one or more contiguous nucleotides) is excised from the genome and the two nucleotides adjacent to the two ends of the excised segment fuse.

*Insertion.* An insertion is an event that occurs when the sequence of one or more nucleotides is added between two adjacent nucleotides in the genome.

*Tandem Duplication.* A tandem duplication is a special insertion event, in which a DNA segment is copied, and then inserted to the position adjacent to itself.

*Inversion.* An inversion is an event that occurs when a continuous nucleotide sequence is inverted in the same position.

*Intra-Chromosomal Translocation (ITX).* An ITX is an event that occurs when a region of nucleotide sequence is translocated to a new position in the same chromosome with inverted orientation.

*Inter-Chromosomal Translocation (CTX).* A CTX is an event that occurs when a region of nucleotide sequence is translocated to a new position in a different chromosome.

Various combinations of the same or different SV types can lead to very complex chromosomal rearrangement events [48]. CNVs, including copy number gains and copy number losses, are generally regarded as a subtype of SVs. NGS-based CNV detection programs use signatures that are quite different from other SV types, and its application in cancer studies has been reviewed elsewhere [49].

#### Breakpoint features

In a typical NGS study, the short sequence reads (~100 nucleotides in length) from a sample genome will be mapped to the reference genome, with SVs detected by identifying unique patterns (or “signatures”) created by the SV events. These diagnostic signatures are connected to the SV breakpoint features, including number of breakpoints, read orientations (also called strands), and coordinate relationships. Here, a breakpoint is a sample genomic position on the two sides of which the base pair coordinates or orientations mapped to a reference genome are not consistent. That is, assuming two continuous base pairs *a_Ns_* and *b_Ns_* in a sample genome have corresponding mapping coordinates *a_Nr_* and *b_Nr_*, with orientations *s_a_* and *s_b_* respectively, in the reference genome, then *a_Ns_* and *b_Ns_* define a breakpoint under any of the following conditions: 1) *b_Nr_* and *a_Nr_* are not on the same chromosome, 2) *b_Nr_* and *a_Nr_* are on the same chromosome but *b_Nr_* − *a_Nr_* ≠ *1* or *S_a_* ≠ *S_b_*. Orientation is the base pair coordinates order in a sample genome relative to reference genome. If the orientation in a sample genome is the same as that in reference genome, it is called “+” direction; otherwise, it is “−” direction. As the direction of a fragment relative to a sample genome is not known, the absolute orientation lacks biological meaning. The orientation only becomes interesting when it flips (from + to –, or from – to +) at a breakpoint, which might be captured in the sequencing data. Each type of SV has its own breakpoint signatures, which are summarized in Figure 1.

### NGS signatures of SVs

As shown in Figure 2, different types of SVs could have different NGS diagnostic signatures across the breakpoints. In this review, we only consider signatures from paired-end sequencing, as single-end sequencing has rarely been adopted in current applications. The accuracy of SV detection depends on the availability of NGS diagnostic signatures of different SV types, which is affected by both the sequencing platform and the alignment tools. Several platforms of NGS have emerged, and some of them are commonly used [14, 15, 50-52]. Likewise, multiple short reads alignment tools have been developed. [53-58]. Different alignment tools or different parameter settings of the same tool will result in different alignment results [59, 60], which will impact the performance of SV detections. There has been a thorough discussion of sequencing platforms and/or alignment tools in literatures. In this section, we focus on the basic elements of NGS signatures for SV detections, which consist of discordant read-pairs and splitting reads.

*Discordant read-pairs.* Since the paired-end NGS technique sequences both ends of each DNA fragment with library insert sizes specific to a given library preparation method and size selection procedure, the two paired reads will be generated at an approximately known distance in the sample genome. A signature of a discordant read-pair is formed when the mapping span and/or orientation of the read-pairs crossing the breakpoint are inconsistent with the reference genome (read-pairs 1 in Figure 2). Specifically, both reads of the pair can be mapped to the reference genome, but they may map to different chromosomes or different orientations, or their coordinates may not agree with the insert size.

*Splitting reads.* A sequence read that spans a SV breakpoint is called a splitting read (see read-pairs 2 in Figure 2). If both splitting parts of a read can be mapped and its mate is uniquely mapped to the reference genome, the splitting read is further masked as a soft-clipped read by some mapping algorithms such as Burrow-Wheeler Alignment(BWA) tool [53]. Otherwise, it is categorized as an un-mapped read. The splitting reads used by current SV detection tools are all soft-clipped reads, and the term “splitting reads” is generally referred as soft-clipped reads. Therefore, a “splitting read” in the following sections refers to soft-clipped read if no further clarification.

Together, discordant read-pairs and splitting reads can corroborate SV events, but they have different inherited strength and weaknesses for certain types of SVs. Generally, discordant read-pairs are more powerful than splitting reads at identifying large SV events, especially ITX and CTX, which are characterized by substantial difference from insert size and/or anomalous orientation. However, it has limited power to determine small SV events, such as small insertion and deletion, which are generally characterized by small deviation from the expected length. Furthermore, the breakpoints of small insertion or inversion events are less likely to be captured by discordant read-pairs. On the other hand, splitting reads for small events can still be mapped to the reference genome as soft-clipped reads or reads with internal gaps, which makes splitting reads more powerful in detecting small deletions, insertions, and inversions. Moreover, splitting reads are able to pinpoint the breakpoint to the nucleotide resolution while discordant read-pairs can only identify the approximate location of breakpoints.

### SV detection programs for WGS data

Sophisticated computational algorithms are crucial to accurately detect SVs from WGS data. Though mostly applied to cancer studies, a number of SV detection programs for NGS data have been developed during the past several years. Here, we describe 9 representative methods including PEMer [16], GASV [17], BreakDancer [4], HYDRA [18], SVDetect [19], CREST [20], DELLY [21], PRISM [22], and LUMPY [37]. They are listed in Table 1 by chronological publishing date. We also applied the selected SV detection programs to tumor–normal Illumina Whole-genome sequencing of a bladder cancer patient. The computing performances, including memory usage and runtime statistics of these SV detection programs, are recorded and summarized in the Supplementary Material of this review. There are other excellent methods available and the methods included here are not exclusive, but they represent a fair survey of commonly used SV callings tools for WGS data.

**Table 1:** A list of selected programs for SV detection using NGS data

Signature reads/read pairs	Method	Publishing Month-Year	Detectable SV type								
			small deletion	large deletion	small insertion	large insertion	small inversion	large inversion	tandem duplication	ITX	CTX
Discordant read pairs	PEMer[16]	Feb-2009		√		√		√			
	GASV[17]	Jun-2009		√		√		√		√	√
	BreakDancer[4]	Sep-2009	√	√	√	√		√		√	√
	HYDRA[18]	Mar-2010	√	√	√	√	√	√	√	√	√
	SVDetect[19]	Jun-2010		√		√		√	√		√
Splitting reads	CREST[20]	Aug-2011	√	√	√	√	√	√		√	√
Discordant read pairs and splitting reads	DELLY[21]	Sep-2012	√	√			√	√	√	√	√
	PRISM[22]	Oct-2012	√	√	√		√	√	√		√
	LUMPY[37]	Jun-2014	√	√		√	√	√	√		√

The selected SV detection programs can be roughly divided into three categories depending on the NGS signatures they used: 1) method based on discordant read-pairs; 2) method based on splitting reads; and 3) method combining discordant read-pairs and splitting reads.

#### Discordant read-pairs based programs

For programs based on discordant read-pairs, we describe PEMer, GASV, BreakDancer, HYDRA, and SVDetect in this section. The discordant read-pairs are usually selected based on program-specific criterion. The common framework of these programs is first to cluster or regroup the discordant read-pairs, with each cluster (usually supported by 3 or more consistent discordant read-pairs) representing a breakpoint or SV event. Then, the SV events are classified by their breakpoint features. Generally, these programs are more powerful in detecting large SV events than small SV events as described before. The only exception is BreakDancer, which designed a special mode for detecting small deletions and insertions with size of 10-100 nucleotides. As the clustering or regrouping of the full set of discordant read-pairs is a NP-complete problem, heuristic methods are necessary for those programs. These programs differ from each other by their means of clustering the discordant read-pairs, as described below.

*PEMer* can detect large insertions, large deletions, and inversions. After the paired-end read-pairs are mapped to a reference genome, read-pairs with abnormal insert sizes, coordinate orders, or orientations are identified as discordant read-pairs. The discordant read-pairs that are likely originating from the same SV are combined into one cluster through a coverage-adjusted multi-cutoff scoring strategy. If there are multiple datasets from different paired-end mapping libraries or from different NGS platforms, the clusters could be merged. PEMer reports the merged clusters and computes statistical significance (i.e., E-values and P-values) for the different types of SVs identified.

*GASV* represents each possible breakpoint region supported by a discordant read-pair as a polygon in a plane, and then it uses a plane-sweeping algorithm to identify the read-pairs that support the same breakpoint by computing intersections of polygons. The type of SV event is then classified by strand orientation and breakpoint coordinates. GASV can also be used for aCGH data, and cluster multiple measurements from different platforms in a single sample.

*BreakDancer* has two modes, BreakDancerMax and BreakDancerMini, with the latter designed for calling insertion and deletions of 10-100 base pairs in size. In BreakDancerMax, the mapped read-pairs in WGS data are first classified into SV types (normal, insertion, deletion, inversion, ITX, and CTX) based on read-pairs separation distances and orientations, user-specified threshold, as well as the empirical insert size distribution estimated from the alignment of each fragment library. The algorithm then searches for genomic regions which anchor more discordant read-pairs than expected on average, and for each region a putative SV is derived from the signatures of the discordant read-pairs. The start and end coordinates are defined as the inner boundaries of the constituent regions that are closest to the predicted breakpoints. A confidence score is also calculated for each putative SV based on a Poisson model that takes into consideration the number of supporting discordant read-pairs, the size of the SV-anchoring region, and the sequencing coverage. BreakDancerMini uses a similar method to predict SVs as BreakDancerMax does, with the exception that BreakDancerMini classifies the read-pairs to normal and discordant pairs using a sliding window test that examines the difference of separation distances between read-pairs that are mapped within the window versus those in the entire genome. This strategy can discover additional discordant read-pairs that are missed by BreakDancerMax. One of the first algorithms developed for SV detection using NGS data, BreakDancer has been used in a number of cancer genome sequencing projects [61-67].

*HYDRA* is designed to localize SV breakpoints from discordant read-pairs by using a heuristic approach. It can detect events including deletions, duplications, inversions, insertions of arbitrary length, and large translocations. It aims to accurately map diverse classes of SVs, including challenging cases involving repetitive elements such as transposons and segmental duplication. It starts by comparing the mappings of discordant read-pairs and identifies collections of discordant read-pairs with consistent patterns. Each collection is a group of discordant read-pairs whose mappings corroborate a common SV event. HYDRA employs a greedy approach to identify a list of SVs from the collections of discordant read-pairs. More specifically, for each putative SV, HYDRA examines the supportive mappings and chooses the single mapping (the “seed”) that is supported by the most other mappings. Subsequent mappings are integrated into the SV call in decreasing order of their overlap with the seed. The breakpoint of a SV is collectively defined as precise as possible by a collection of discordant read-pairs. HYDRA usually does not classify variants or group multiple breakpoints into a single variant call, which reduces assumptions about variant structure and increases sensitivity, but necessitates a subsequent classification step.

*SVDetect* can detect large insertion, large deletion, inversion, tandem duplication, and CTX. Similar to other programs using discordant read-pairs, the first step in SVDetect is to regroup all pairs that are suspected to originate from the same SV. It then uses a sliding-window strategy to identify groups of discordant pairs sharing a similar genomic location, and each pair of these genomic location windows is called a “link”. The identified links are filtered by using user-defined parameters such as minimum number of discordant read-pairs supporting a link, and the filtered links are clustered by their orientations and order of supporting reads, and insert sizes. The SV types of these clusters are predicted based on the breakpoint signatures.

With respect to somatic SV detections in cancer genomes, GASV, BreakDancer, and SVDetect can compare SVs across multiple samples, and can call somatic SVs directly from tumor and matched normal samples. On the other hand, the current versions of PEMer and HYDRA do not have the functionality to call somatic SVs directly from tumor and matched normal WGS data, and requires a post processing step to eliminate germline SVs from tumor for somatic SV detection.

#### Splitting reads based programs

As a representative program solely based on splitting reads to determine the positions of somatic SV breakpoints, CREST is the focus of this section. CREST identifies the first part of a breakpoint by the presence of splitting reads, and then detects its partner by an assembly-mapping-searching-assembly-alignment procedure. This procedure includes the following steps: 1) assembling the unaligned portions of splitting reads clustered to the first part of a breakpoint to determine a contig (which is the sequence of the longest unaligned portion); 2) mapping the contig to the reference genome and searching the possible positions for the second part of the breakpoint; 3) assembling the unaligned portions of soft-clipped reads clustered to the second part of a breakpoint to determine another contig; 4) aligning the second contig to the reference genome to see whether it confirms the position of the breakpoint's first part. If both parts of the breakpoint are confirmed, CREST classifies SV event by the signatures in orientations and breakpoint coordinates.

By using a splitting read signature, CREST can pinpoint the breakpoints of SVs to nucleotide resolution. Furthermore, as this algorithm uses information from both sides of a breakpoint to double check its accuracy, the false positive rate at detecting breakpoints is low, especially in regions with high mapping rates. Applying CREST to a human melanoma cell line identified 160 somatic SVs, and over 80% of them were validated by Sanger sequencing [20]. CREST has been adopted in a number of cancer genome sequencing projects [68-80]. The false positive calls are usually coming from mis-alignment, which could be reduced by manual review of the aligned splitting reads at the breakpoints. Specially designed for detecting somatic SVs, CREST can filter out germline events with overlapped splitting reads in tumor and normal WGS data. SVs in regions with low mappability, however, pose major challenges for CREST. Since it examines the whole reference genome for mapping the unaligned portion of soft clipped reads, the current version of CREST is relatively time consuming and memory demanding.

#### Programs combining discordant read-pairs and splitting reads

For programs combining discordant read-pairs and splitting reads, we describe DELLY, PRISM and LUMPY in this review. DELLY and PRISM use the two types of signatures in a stepwise manner, while LUMPY uses the two types of signatures in parallel and integrates the results by a probabilistic method. More specifically, DELLY and PRISM first cluster discordant read-pairs to determine SVs, and then refine the results with splitting reads to reach single-nucleotide breakpoint resolution by using a specially designed aligner. The two programs differ in the way of clustering discordant read-pairs and aligning splitting reads. LUMPY aligns discordant read-pairs and splitting reads independently, determines the breakpoint position intervals with probability at each position, and then clusters the overlapping intervals and integrates their probabilities to determine the SV types and breakpoints.

*DELLY* sorts and bins the discordant read-pairs into an undirected, weighted graph which groups read-pairs supporting the same SV based on their orientations and coordinates. Each type of SV is analyzed separately and thus each deletion, inversion, tandem duplication, and translocation can be nested into a single complex event. DELLY does not support insertion detection. Following the discordant read-pairs analysis that identify breakpoint containing genomic intervals, the splitting reads analysis can refine the breakpoint to nucleotide resolution using a fast k-mer-based alignment algorithm. A special version of banded alignment is implemented by combining the alignment results from SV-containing reference regions, with the breakpoint determined by selecting the position that gives the highest combined score. Since its release, *DELLY* has been used in several cancer genome sequencing projects [77, 78, 81-83]

*PRISM* starts with identifying discordant read-pairs and splitting reads. The discordant read-pairs are clustered using a greedy algorithm that groups together pairs with similar mapping distance and orientation. Then, it uses a modified Needleman-Wunsch (NW) algorithm for split mapping of splitting reads. In the split mapping step, it first tries to align splitting reads in the concordant region, allowing for one insertion or deletion with fixed penalty. If there are discordant clusters within the concordant region, the splitting reads is aligned in a way that allows one part of it to map to the concordant region and the other part to the discordant region. Lastly, PRISM calls the SV loci and filters the initial list of SVs based on the number of supporting reads and the alignment score, with an option to set thresholds for sensitivity and specificity. PRISM is able to detect multiple SV types, including arbitrary-sized inversions, arbitrary-sized deletions, small insertions, and tandem duplications. The authors also developed a tool called PRISM-CTX to call CTX.

*LUMPY* provides a framework based upon a general probabilistic representation of an SV breakpoint that allows any number of alignment signals to be integrated into a single discovery process. While the major types of signals are alignment of discordant read-pairs and splitting reads, other signals such as read depth calls and prior knowledge of breakpoint can also be incorporated. LUMPY aligns the discordant read-pairs and determines a pair of intervals upstream or downstream the mapped reads for possible breakpoint positions. The size of the intervals and the probability of observing a breakpoint at each position are based on the empirical size of the sample's fragment library. LUMPY considers splitting reads with two or more splitting parts. It aligns each splitting part of a read to the reference genome, and aligns the adjacent splitting part to non-adjacent locations in the reference genome. To account for the possible errors in sequencing and alignment, each alignment pair maps to two breakpoint intervals centered at the middle point and decrease exponentially toward their edge. The size of the interval is a configurable parameter and is based on the quality of the sample and the specificity of the alignment algorithm. Once the evidence from different alignment signals is mapped to the breakpoint intervals, overlap intervals are clustered and the probabilities are integrated. A key difference between LUMPY and DELLY/PRISM is that it simultaneously instead of sequentially integrates the multiple SV detection signals during SV discovery. Any clustered breakpoint region that contains sufficient evidence (user-defined argument) is returned as a predicted SV. The SV types identified by LUMPY include deletions, inversions, tandem duplications, and CTX. Identification of small insertions that are spanned by a discordant read-pair or contained by a splitting read is not explicitly supported by LUMPY, and a post-processing step is required.

In terms of somatic SV detections in cancer study, DELLY and LUMPY can compare SVs across multiple samples, and can call somatic SVs directly from tumor and matched normal samples. A post processing step to eliminate germline SVs is required for PRISM, the current version of which does not have the mode to directly call somatic SVs from tumor and matched normal WGS data.

### Challenges

While a number of computational tools have been developed for NGS-based SV calling in the cancer genome, none of them is comprehensive enough to include all SV types and reconstruct all the SV events at high accuracy. There are still many challenges in somatic SV detection, which are introduced by the limitations of NGS technologies, complexities of tumor samples, and difficulties of SV event reconstruction and SV mechanism inference.

#### Limitations of NGS technologies

While NGS has provided unprecedented power in SV detection as aforementioned, the short read length data generated also introduces the issue of read mapping ambiguity. This is especially problematic for reads from repetitive regions [84], which are known to be SV hotspots [21, 85]. When a read in a discordant pair or a part of a splitting read can be mapped to multiple locations in the reference genome, it becomes challenging to determine where the corresponding SV is from. While it might be possible to report multiple SV candidates with varying confidence scores, it will bring additional burdens for Sanger validation by including more potential false calls. Soft clustering, which allows the use of mate pairs with multiple good mappings, has been used to improve SV detection performance [86]. Many of the latest mapping programs have options to select the best mapped reads and to manage suboptimal ones, but few existing SV detection methods take full advantage of all the information available.

**Figure 3:**
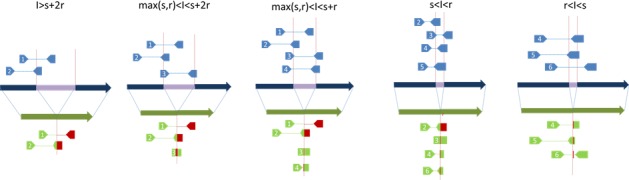
An exemplary illustration of the impact of SV event sizes and library insert sizes on the NGS signatures I: length of insertion event (purple strand); r: read length; s: length of un-sequenced part in a read-pair; insert size equals 2r+s, assuming reads are in same length.

The paired-end sequencing strategy commonly adopted in NGS technology provides an alternative way to increase effective read length and mapping accuracy. One of the major advantages of paired-end sequencing is that the mapping of one end will aid and improve the mapping quality of the other end. However, paired-end sequencing introduces additional issues such as the wide range of insert size for a specific sequencing library and the increase in cost associated when using multiple library sizes. Smaller insertion and deletion events introduce discordant reads that are often missed. Even if larger insert sizes and multiple sequencing libraries are used, the issue of multiple mappings remains for longer repeat elements that number in the millions in the human genome. Due to the countless combinations of possible SV event sizes and library insert sizes (Figure 3), and the fact that many complex SV events exist, some splitting reads may not be mapped. Therefore, the ability of short reads and read-pairs generated by NGS to accurately capture SV signatures relies on multiple factors, including the type, size and location of a SV event, the library insert size distributions, the mapping algorithms, and the chance of signature reads being mapped to correct reference position.

A commonly used strategy for improving SV detection is to use deeper coverage to compensate for shorter reads, as the accuracy of break-point detection will improve with increasing read depth. However, the nature of short read length of current NGS technologies poises challenges that coverage depth cannot always overcome, especially for SVs in low complexity regions. These limitations might be overcome by longer sequence reads generated from new sequencing technologies, such as Single Molecule Real Time (SMRT) sequencing from Pacific Biosciences (PacBio) [87]. While the current SMRT sequencing platform has higher sequence error rates, the long reads generated by this platform has provided tremendous advantages [88] in hybrid (correcting read errors by short reads) [89] or non-hybrid [90] *de novo* genome assemblies and in resolving genome complexities including SVs [91]. Recently, PacBio released their new P6-C4 chemistry that significantly decreases error rates and expands sequence length (median length >14 kb). The continuing improvements in sequencing technologies and their adoption in cancer genome sequencing are expected to improve our capacity to detect somatic SVs.

#### Complexities of tumor samples

Another challenge in somatic SV detection comes from the complexities of tumor samples, including tumor purity and heterogeneity. Tumor samples are inevitably contaminated by normal tissues of unknown fraction. Tumor sample could contain multiple sub-clones that evolve due to tumor progression or tumor stem cell populations, and sub-clones are important to tumor evolution and cancer relapse [3]. With normal tissue intermixed in a tumor sample, the portion of signature reads supporting a somatic SV event is diminished, along with the number of supporting discordant read-pairs or splitting reads. This issue will need to be considered in both study design [92, 93] and data analysis stages in order to achieve improved detection sensitivity and specificity. For example, SV detection programs usually specify the cutoff numbers of signature reads (e.g., CREST and DELLY) or score the supporting evidence (e.g., LUMPY) to make a detection call. An accurate cutoff or score will depend on tumor cell percentage and the sequencing read depth of the investigated sample. As the tumor purity in different samples might vary greatly and are often unknown, it is challenging to determine the proper cutoff/score for accurate SV calling. Likewise, the SV events from a minor clone are difficult to identify due to the diminished signature reads. Some signature reads from a minor clone might even be filtered out as noise in the processing step. While increasing the sequencing depth can help capturing low-purity tumor SVs and/or sub-clonal SVs, the cost could become an issue in practice as higher coverage of sequencing inevitably requires higher costs. Furthermore, the sequencing coverage is not uniformly distributed across the genome, which creates substantial difficulties for SVs in regions with lower coverage.

#### Complexity of tumor genome

Compared with germline sample, tumor samples often display very different and highly rearranged genomes, resulting in complicated SVs [48, 94], which are hard to decipher. Complicated SV events are a series of SVs that happen within a small genomic range such that some of the signatures of those events are removed. Therefore, complicated SV event inference cannot be solely based on single breakpoint, and a comprehensive analysis of all breakpoints is necessary. For example, chromothripsis is a phenomenon in the cancer genome [94-97] that features massive inter-chromosome translocations between several chromosomes and confined segmental copy number status. Detection and inference of chromothripsis is still in the early stage of development with few analysis methods available [98, 99]. As complicated SV events most likely happen in a stepwise manner, the study of cancer genome evolution [3, 100-103] might help to reconstruct the SV events.

#### Reconstruction and Validation of SV events

Most SV detection methods identify breakpoints using the signatures mentioned in previous section and infer the SV types by breakpoint signatures. When a SV event has multiple breakpoints, those breakpoints could be characterized independently by splitting reads, discordant read-pairs, or both. Some breakpoint features are unique to a specific type of SV event, while others are shared by multiple events. For example, one of the two breakpoints in an insertion event with an inserted DNA segment from the same chromosome has a unique signature, while the other one has the same signature as the breakpoint of a deletion event (Figure 1). Furthermore, an insertion event with an inserted DNA segment from another chromosome may initially be identified as two CTX events. After characterizing all breakpoints, a post-processing step is necessary to infer the SV events that generate those breakpoints. This procedure is generally lacking in existing SV detection methods. Furthermore, the intermixture of breakpoints from major and minor clones of a heterogeneous sample creates tremendous troubles in SV event inference. Given an imperfect list of SVs or breakpoints, reconstruction of the underlying chromosome or genome structure remains a great challenge.

Often an orthogonal experimental method is needed to validate predicted SVs from NGS data, and the commonly used approach is PCR amplification followed by Sanger Sequencing [20, 21, 39]. PCR amplification can confirm larger size events (bounded by maximum amplicon size) [21, 39] with carefully designed primers. The failure of PCR reactions may not reject the existences of SV candidates, as the failures might be caused by sequence-specific experimental conditions such as thermocycle or primer designs. After PCR, Sanger sequencing is employed as the approach in validating SV breakpoints [20] and small insertions and deletions [39] at nucleotide resolution. When multiple breakpoints are within the range of Sanger sequencing, inference of complex SV events might be achieved by designing multiple groups of primer pairs corresponding to all possible events combinations. However, the aforementioned difficulty of reconstruction of SV events from a list of breakpoints also creates substantial challenges in confirming the predicted SV events. Furthermore, the increased costs of reagents, labor, and time in those experiments will set limits to the amount of SV candidates that can be evaluated. Therefore, in practice only a selected portion of predicted SV candidates will subject to validation.

As an alternative approach, one can merge SV calls from multiple programs based on the assumption that common calls could raise their confidence level and increase overall sensitivity. Due to the aforementioned difficulties in reconstruction of SV events, it is more feasible to compare the predicted breakpoints from different tools, rather than to evaluate the predicted SV events. Since the programs based on discordant read pairs and splitting reads have different power in pinpointing the breakpoints, one can allow some margins (for example, ±50 nucleotides) for two predicted breakpoints to be considered as concordant. It should be noted that the standard for evaluating SV calls from different programs is generally lacking, and there is a need for the community to have the rules set.

#### Inference of SV mechanisms

SVs might be triggered by replication or transcription errors, genotoxic or oxidative stress, or combinations of these [104]. Three main types of mechanisms are recognized to cause SVs [8, 105], including non-allelic homologous recombination (NAHR), non-replicative non-homologous repair, and replication-based mechanisms. While it remains challenging to infer SV mechanisms in the cancer genome, a closer examination of identified SVs can help to understand the underlying mechanism. For example, a SV in a region with loss of heterozygosity is likely caused be NAHR; SVs involving repetitive and transposable elements are likely caused by retro-transposition and microhomology-mediated break-induced replication [106]; and ITX and CTX may result from random non-homologous end joining of fragments after chromothripsis [94]. Conversely, having prior knowledge of a SV mechanism would aid in selecting the best possible SV event from the candidates, and therefore improve the analysis accuracy in both SV detection and event reconstruction.

## CONCLUSIONS AND OUTLOOKS

The revolutionary advances of NGS technologies and their growing adoption in cancer research have made it possible to screen for somatic variations in cancer genomes on an unprecedented scale. As one of the most clinically important somatic aberrations, SVs in tumor genomes is believed to have high probability of harboring oncotargets. Sophisticated computational tools are required to couple with NGS methodologies to accurately detect somatic SVs from the massive amount of raw data generated for each sample. During the past several years, a number of computational methods have been developed to identify SVs based on their NGS signature. Each method has its own unique limitations and strengths, such as read mapping or clustering strategy, use of discordant read-pairs and/or splitting reads, and focus on certain types of SVs. In this article, we reviewed nine methods to provide a guide of the analytical tools developed in this research field.

Despite unprecedented progress in our ability to map and analyze SVs in the cancer genome, accurate and complete detection of somatic SVs remains challenging and we are far from understanding the causes and consequences of the SVs that are observed [107]. The challenges caused by the limitations of NGS technologies, complexities of tumor samples, difficulties of SV event reconstruction and SV mechanism inference remain. Nevertheless, the last 5 years has witnessed tremendous advances in this exciting field, and we expect new analysis methods built on improved sequencing techniques will be developed to tackle these challenges and provide better SV detection.

## SUPPLEMENTARY MATERIAL TABLES


