# Supplementary material for: Structural variation discovery in the cancer genome using next generation sequencing: Computational solutions and perspectives
Source: Oncotarget. 2015 Mar 8;6(8):5477–89. doi: 10.18632/oncotarget.3491 (PMC4467381; doi:10.18632/oncotarget.3491)
Supplement: Supplementary file 1 [file oncotarget-06-5477-s001.pdf]

Structural variation discovery in the cancer genome using next generation sequencing: Computational solutions and perspectives

Supplementary Material

Evaluating computing performance of SV detection programs for WGS data

In this section, we apply eight SV detection programs to tumor–normal Illumina WGS of a bladder cancer patient. The computing performances, including maximum memory usage and runtime statistics of these SV detection programs, are recorded and summarized.

The tested pair-end WGS dataset was generated by Illumina sequencing service with Illumina HiSeq 2000 platform on the primary tumor and matched blood of a patient with muscle-invasive transitional cell carcinoma of the urinary bladder (TCC-UB). The read length of each end is 100 nucleotides, and the mean library insert sizes are 320±15 nucleotides and 313±15 nucleotides for tumor and matched normal samples, respectively. Sample preprocessing, fragmentation, and library preparation was performed following Illumina's standard protocols. The raw image data was processed by CASAVA [1], and the sequence reads mapped to the hg19 reference genome using BWA [2]. The mean coverages are 61x and 46x, and the sizes of the resulted BAM files are 155 and 119 GBs for tumor and matched blood, respectively.

The tested SV detection programs include GASV [3], BreakDancer [4, 5], HYDRA [6], SVDetect [7], CREST [8], DELLY [9], PRISM [10], and LUMPY [11]. PEMer [12] was not included due to the extremely high computational demand of its MEGABLAST [13] mapping step. The BAM files generated by BWA mapping serve as the inputs for all tested programs except for PRISM which requires SAM format. The programs that support parallel computing are tested under a computer cluster composed of 100 nodes and each node has two Intel Xeon E5-2670 @2.6 GHz processors and 64 GB of memory. The programs that do not support parallel computing are tested using a Dell Linux workstation with two Intel Xeon E5-2620 v2 @2.1 GHz processors and 32 GB of memory.

Supplemental Table 1 showed the performance statistics for the programs that support parallel computing setting. Different programs support parallel computing in different ways. For examples, CREST splits the jobs by chromosomes, DELLY calls different types of SVs in parallel, and SVDetect and LUMPY use pre-specified multithread in computation. The default or recommended settings are used for each program except for HYDRA. As the realignment component of HYDRA utilizes novoalign [14] whose non-commercial version doesn't support multithread in computation, we split the reads files into small files with each containing 100,000 reads during its realignment. As shown in Table S1, all programs except for SVDetect are finished within 2 days in our testing computer cluster. The computing statistics of the programs that do not support parallel computing are listed in Supplemental Table 2. The default settings are used for all these programs. They all finish within several hours in our testing workstation, with GASV using much more memory (9GB) than other two (1GB).

It should be emphasized that many additional factors such as data quality, complexity of cancer genome, and sequencing coverage, could affect the statistics of memory usage and runtime. Furthermore, these factors may affect the computing performances of different SV detection programs to different extents. For example, increased number of splitting reads will greatly slow down the programs requiring reads realignment, but might have relatively less effect on the run times of the programs without realignment step. A systematic study of the performance of each method is beyond the scope of this review.

Supplemental Table 1: The runtime and memory usage of selected multiple-thread programs for somatic SV detection in a WGS tumor-normal pair

Programs[a]	Realignment	Available multi-process mode	Multi-process setting[b]	Memory usage[c] (GB)	Runtime[d] (hours)
HYDRA	Novoalign	Splitting reads file into multiple small files for Novoalign mapping	In the two Novoalign steps, the reads file is split into smaller units (462 for step 1, and 230 for step 2) with each containing 100,000 reads	33.2	23
SVDetect	No realignment	Computing with multiple threads	12 threads	12.2	>370
CREST	BLAT	Splitting the job to multiple tasks by chromosomes	23 tasks by chromosomes	6.1	6
DELLY	Integrated	Splitting the job to multiple tasks by SV types; computing with multiple threads by number of samples	4 tasks (insertion, duplication, inversion, and translocation) ; 2 threads	1.2	27
LUMPY	YAHA	Computing with multiple threads in YAHA mapping step	20 threads	12.9	30

a. Analysis was performed in a Linux cluster composed of 100 nodes with two Intel Xeon E5-2670 @2.6 GHz processors and 64 GB of memory for each; b. Default settings were used for all programs except for HYDRA, as the commercial version of its realignment program (Novoalign) is not available to us; c. The maximum memory usage in any step of the SV calling; d. The sum of elapsed time for the most time consuming task in each step is used to estimate the run time.

Supplemental Table 2: The runtime and memory usage of selected single-thread programs for somatic SV detection in a WGS tumor-normal pair

Programs[a]	Realignment	Memory usage (GB)[b]	Total runtime (hours)
GASV	No realignment	9.1	6
BreakDancer	No realignment	0.8	5.5
PRISM	Integrated Needleman-Wunsch algorithm	1.0	4

a. The computational environment is a DELL Linux workstation with two Intel Xeon E5-2620 v2 @2.1 GHz processors and 32 GB of memory, and the default settings were used for all programs; b. The maximum memory usage in any step of the SV calling steps.

1. http://support.illumina.com/sequencing/sequencing_software/casava.html.
2. Li, H. and R. Durbin, *Fast and accurate short read alignment with Burrows-Wheeler transform.* Bioinformatics, 2009. **25**(14): p. 1754-60.
3. Sindi, S., et al., *A geometric approach for classification and comparison of structural variants.* Bioinformatics, 2009. **25**(12): p. i222-30.
4. Chen, K., et al., *BreakDancer: an algorithm for high-resolution mapping of genomic structural variation.* Nat Methods, 2009. **6**(9): p. 677-81.
5. Fan, X., et al., *BreakDancer - Identification of Genomic Structural Variation from Paired-End Read Mapping.* Curr Protoc Bioinformatics, 2014. **2014**.
6. Quinlan, A.R., et al., *Genome-wide mapping and assembly of structural variant breakpoints in the mouse genome.* Genome Res, 2010. **20**(5): p. 623-35.
7. Zeitouni, B., et al., *SVDetect: a tool to identify genomic structural variations from paired-end and mate-pair sequencing data.* Bioinformatics, 2010. **26**(15): p. 1895-6.
8. Wang, J., et al., *CREST maps somatic structural variation in cancer genomes with base-pair resolution.* Nat Methods, 2011. **8**(8): p. 652-4.
9. Rausch, T., et al., *DELLY: structural variant discovery by integrated paired-end and split-read analysis.* Bioinformatics, 2012. **28**(18): p. i333-i339.
10. Jiang, Y., Y. Wang, and M. Brudno, *PRISM: pair-read informed split-read mapping for base-pair level detection of insertion, deletion and structural variants.* Bioinformatics, 2012. **28**(20): p. 2576-83.
11. Layer, R.M., et al., *LUMPY: a probabilistic framework for structural variant discovery.* Genome Biol, 2014. **15**(6): p. R84.
12. Korbel, J.O., et al., *PEMer: a computational framework with simulation-based error models for inferring genomic structural variants from massive paired-end sequencing data.* Genome Biol, 2009. **10**(2): p. R23.
13. Zhang, Z., et al., *A greedy algorithm for aligning DNA sequences.* J Comput Biol, 2000. **7**(1-2): p. 203-14.
14. http://www.novocraft.com/main/page.php?s=novoalign.
